# Should we despecialize the training? Postgraduate training in psychiatry in the time of the COVID-19 outbreak in poland: Challenges & solutions

**DOI:** 10.1192/j.eurpsy.2021.2200

**Published:** 2021-08-13

**Authors:** A. Szczegielniak, T. Gondek, A. Rewekant

**Affiliations:** 1 Department Of Psychiatric Rehabilitation, Medical University of Silesia in Katowice, Katowice, Poland; 2 Early Career Psychiatrists Committee, European Psychiatric Association, Wroclaw, Poland; 3 General Psychiatry Unit I, Greater Poland Neuropsychiatric Center, Kościan, Poland

**Keywords:** Postgraduate training, COVID-19, Medical Education

## Abstract

**Introduction:**

The outbreak of the SARS-CoV-2 epidemic forced a change in the functioning of health care systems across the globe, requiring rapid adaptation to new conditions for the safe provision of services within all medical fields. General disruption has also affected the traditional program of a postgraduate training, which has been so far fixed with temporary solutions, but not given a proper evaluation in the times of big expectations and pressures from both patients and healthcare workers.

**Objectives:**

Outbreak of the COVID-19 put psychiatry trainees and Early Career Psychiatrists in an unprecedented position of responsibility for treatment of a variety of comorbidities they had no prior experience with due to closure of specialized hospital departments and limited access to regular diagnostic tools. In addition to changes in clinical practice and deployment to unfamiliar ground, rescheduling of different components of regular training, transferring most of the educational activities to distance learning, limiting professional growth by canceling most courses and conferences only strengthened the feeling of uncertainty caused by constant adjustments of the final examinations’ conditions.

**Methods:**

The Speciality Training Section of the Polish Psychiatric Association decided to review changes forced by the COVID-19 outbreak in a traditional postgraduate training program in psychiatry.

**Results:**

Identified shortcomings pose questions about the necessity of a solid revision of the training in order to cope with more demanding working conditions.

**Conclusions:**

Presented recommendations may be the starting point for a discussion on the programs’ evaluation across the entire region.

**Disclosure:**

No significant relationships.

